# Interaction of *Treponema pallidum*, the syphilis spirochete, with human platelets

**DOI:** 10.1371/journal.pone.0210902

**Published:** 2019-01-18

**Authors:** Brigette Church, Erika Wall, John R. Webb, Caroline E. Cameron

**Affiliations:** 1 Department of Biochemistry and Microbiology, University of Victoria, Victoria, BC, Canada; 2 Trev and Joyce Deeley Research Centre, British Columbia Cancer Agency, Victoria, BC, Canada; Monash University, AUSTRALIA

## Abstract

Extracellular bacteria that spread via the vasculature employ invasive mechanisms that mirror those of metastatic tumor cells, including intravasation into the bloodstream and survival during hematogenous dissemination, arrestation despite blood flow, and extravasation into distant tissue sites. Several invasive bacteria have been shown to exploit normal platelet function during infection. Due to their inherent ability to interact with and influence other cell types, platelets play a critical role in alteration of endothelial barrier permeability, and their role in cancer metastasis has been well established. The highly invasive bacterium and causative agent of syphilis, *Treponema pallidum* subspecies *pallidum*, readily crosses the endothelial, blood-brain and placental barriers. However, the mechanisms underlying this unusual and important aspect of *T*. *pallidum* pathogenesis are incompletely understood. In this study we use darkfield microscopy in combination with flow cytometry to establish that *T*. *pallidum* interacts with platelets. We also investigate the dynamics of this interaction and show *T*. *pallidum* is able to activate platelets and preferentially interacts with activated platelets. Platelet-interacting treponemes consistently exhibit altered kinematic (movement) parameters compared to free treponemes, and *T*. *pallidum*-platelet interactions are reversible. This study provides insight into host cell interactions at play during *T*. *pallidum* infection and suggests that *T*. *pallidum* may exploit platelet function to aid in establishment of disseminated infection.

## Introduction

Syphilis, caused by the spirochete *Treponema pallidum* subsp. *pallidum* (hereafter *T*. *pallidum*), is a chronic, sexually transmitted infection affecting an estimated 36 million people worldwide, with 11 million new cases occurring annually [1,2]. In recent years rates of primary and secondary syphilis have risen sharply in particular populations, most prominently amongst men who have sex with men, while a general increase in infectious syphilis cases in both heterosexual men and women has been observed in cities across North America, Europe, and Asia [1,3,4]. Paralleling the rise in syphilis cases amongst women, congenital syphilis cases have increased in middle and high income countries and continue to be a leading cause of stillbirth in low income nations, affecting an estimated 1.36 million pregnant women each year and resulting in over 500,000 adverse outcomes from maternal syphilis [5].

Syphilis is a multistage disease punctuated by asymptomatic periods of latency. The primary and secondary stages of syphilis present with a painless chancre at the initial site of infection followed by a non-pruritic rash, respectively, both of which spontaneously resolve [2]. Treponemes rapidly disseminate from the initial site of infection via the circulatory system, with invasion of the central nervous system occurring in up to 40% of early infections [6]. Indeed, neurological symptoms such as stroke may occur at any stage of infection, as can ocular involvement which is often accompanied by abnormal cerebrospinal fluid (CSF) [7–10]. In the absence of antibiotic treatment, the majority of infected individuals exhibit lifelong latency while approximately 30% of patients exhibit symptoms of tertiary syphilis, which can include gummatous lesions, neurosyphilis and/or cardiovascular syphilis [2,11,12].

Circulating near the blood vessel walls as flattened disks, inactive platelets are optimally situated to rapidly respond to deviations in the vascular environment and perform central roles in hemostasis and in the modulation of inflammation, immune responses and endothelial permeability [13]. Ligand binding to platelet receptors induces specific signaling and secretion of granule components [14,15]. Cytoskeletal restructuring occurs and the platelet transforms from the inactive state to the active spheroid state, which becomes spherical and characterized by pseudopod extensions and up-regulation of key receptors on the platelet surface including P-Selectin, αIIbβ3 integrin and LAMP-3 (lysosomal-associated membrane protein 3) [13]. Platelets tether to the endothelium via the pseudopods and may further activate to a spread fully activated form [13]. Platelet aggregates may be composed of both spheroid and spread platelets [16]. Activated platelets have been shown to loosen endothelial cell junctions [17] and promote leukocyte recruitment via secreted platelet chemokines [18]. Responding leukocytes localize to these foci of increased endothelial permeability and extravasate. This strategy has been shown to be exploited by tumor cells to promote perivascular infiltration [19–21].

Platelets have also been shown to be a target of several invasive pathogens including *Staphylococcus aureus*, *Streptococcus pyogenes* and *Borrelia* species [22–27]. Upon pathogen interaction, platelets can become activated by direct or indirect receptor contact with bacterial virulence factors, with the latter occurring via a bridging plasma protein [28]. Further, platelets have been shown to promote *S*. *pyogenes* dissemination in a murine sepsis model, where significantly fewer bacteria disseminated to the blood, lungs and spleen in platelet-depleted animals [29]. Surprisingly, despite the potential importance of platelet interactions in bacterial pathogenesis, pathogen-platelet investigations remain a relatively unexplored field of study.

*Treponema pallidum* is an obligate human pathogen that can rapidly invade the circulatory system and traverse the blood-placenta, blood-retina and blood-brain barriers [2]. This invasive capacity, together with the known ability of several other invasive pathogenic bacteria to target platelets, prompted us to investigate whether platelet exploitation may also play a role during *T*. *pallidum* infection. Previous studies have determined that *T*. *pallidum* binds a variety of nucleated mammalian cells in culture [2,30–32], but to date no studies have been undertaken to investigate the capacity of *T*. *pallidum* to interact with platelets. Disease symptoms associated with syphilis that are consistent with potential *T*. *pallidum*-platelet interactions include occlusive stroke, congenital thrombocytopenia, and aortic aneurism [7,33–35].

In this study we investigate the potential interaction of *T*. *pallidum* with human platelets using a modified method of darkfield video microscopy to compile high resolution datasets of live treponemes with fresh human platelets and flow cytometry to quantitate treponeme-platelet interactions and determine the dependence of this interaction upon treponeme viability. Herein we demonstrate that *T*. *pallidum* interacts with platelets in a manner that correlates with the degree of platelet activation. We investigate treponeme-platelet interactions during stationary adhesion and show that *T*. *pallidum* displays phenotypic changes during platelet interactions by forming a more compact shape and by increasing its axial rotation and the velocity of its translational motility. We also demonstrate that platelet-tethered treponemes exhibit reduced displacement under the force of moving plasma and that treponemes are able to induce platelet activation. This study may reveal a role for treponeme-platelet interactions in *T*. *pallidum* pathogenesis.

## Materials and methods

### Ethics statement

All human blood studies were approved by the local Institutional Review Board at the University of Victoria and samples were obtained by informed consent from volunteer donors. All animal studies were approved by the local Institutional Review Board at the University of Victoria and were conducted in strict accordance with standard accepted principles as set forth by the Canadian Council on Animal Care, National Institutes of Health and the United States Department of Agriculture in a facility accredited by the Canadian Council on Animal Care and the American Association for the Accreditation of Laboratory Animal Care.

### *T*. *pallidum* propagation and extraction

Propagation and harvesting of *T*. *pallidum* was performed as per Lukehart and Marra [36], with the exception that testicular extractions were performed under microaerophilic conditions of ~5% oxygen in a Coy Laboratory Products anaerobic chamber (Mandel Scientific Company Inc., Guelph, ON, Canada) to enhance *T*. *pallidum* viability [11,37,38].

### *T*. *pallidum* heat treatment

A subset of viable treponemes were incubated at 56˚C for 45 minutes to abrogate motility [39,40]. Heat-treated treponemes were then assessed by darkfield microscopy to ensure they were non-motile yet remained morphologically consistent with viable treponemes.

### Platelet purification

Donor blood was extracted from healthy volunteers into BD Vacutainers ACD-A tubes (BD Canada, Mississauga, ON), transferred into sterile 15 mL conical tubes (Sarstedt Inc., Montreal, QC) and centrifuged at 180 x *g* for 15 minutes at 22˚C (no acceleration or brake). The platelet rich plasma (PRP) portion was transferred, with care not to disturb the buffy coat, into fresh conical tubes, and allowed to slowly run down the inside of the tube. This process yielded an average of 5.0 x 10^5^ platelets/μL. Platelet poor plasma (PPP) was obtained from the supernatant of PRP centrifuged at 15,000 x *g* for 15 minutes. All PRP and PPP samples were stored at room temperature. Platelet activation stages were assigned as described in Zhao et al. [41].

### *T*. *pallidum*-platelet co-incubation

All co-incubations of treponemes and platelets were initiated in a microaerophilic chamber (5% oxygen). Samples were then either maintained at 34˚C and 5% oxygen or tightly sealed and transferred to co-incubate at 37˚C in atmospheric oxygen. All live treponeme treatments, such as staining or fixation, occurred only under microaerophilic conditions.

### Darkfield microscopy

To achieve optimal magnification and high resolution, the *T*. *pallidum*-platelet sample volume was limited to 4 μL per 1.0–1.2 mm thick glass slide with a 22 x 36 mm cover glass (Fisher Scientific Company, Ottawa, ON) gently pressed into place. Slides were viewed at 1000x oil magnification with a 100x oil pan-fluor objective lens set to 0.7 on a Nikon Eclipse 80i darkfield microscope with a Nikon DS-Qi1Mc digital camera with NIS-Elements imaging software (Nikon Canada Inc., Mississauga, ON). For video microscopy (high resolution real-time imaging), exposure times were set at 3–40 ms. For additional brightness, gain was increased to 9–16. Image clarity was directly related to the level of platelet activation and platelet microparticle (PMP) secretion. As activation and PMP secretion increased, additional light was scattered, resulting in a brighter field, and exposure times were further reduced to compensate. The quality of imaging samples with reduced light scattering was improved by increasing the exposure time to ≥ 60 ms and opting for still imaging rather than videos.

### Live treponeme and platelet analysis

Treponeme motility and platelet interactions were monitored by darkfield microscopy. For field of view (FOV) counts, 20 random fields per slide were counted. Treponeme viability was associated with vigorous to moderate activity consisting of axial rotation, flexing, bending and translational motility (forward and backward motion). Platelet sub-populations spontaneously adhere to glass slides during microscopic observation; the number of treponemes bound to slide-adhered platelets were enumerated as above. Treponeme speed and relative displacement in plasma currents were calculated from microscopy video segments obtained prior to the plasma reaching steady state.

### Image and video acquisition and analysis

Micrographs and videos were captured with a Nikon DS-Qi1Mc digital camera (Nikon Camera Inc.). Images were saved in uncompressed JPEG format or as AVI movies. ImageJ (ImageJ v1.6.0_24/1.51h, embedded in Fiji) [42,43] and GIMP software (GIMP 2.8.18, http://www.gimp.org/) were used to adjust brightness and contrast, scale, dpi and sharpness. Contrast in multi-panel images was harmonized during figure construction in Excel 2016 (version 1806, Microsoft, Redmond, WA). For optimum definition of the treponeme to measure wavelength, amplitude and bacterial cell length, images were imported into Excel, enlarged to 400% and contrast-enhanced. Images were selected with all or the majority of the treponeme flat-wave in the imaging plane [44]. Wavelengths were measured from peak apex to peak apex and averaged. Amplitude was measured from peak apex to trough and divided by two at five separate points along the cell body and averaged per treponeme. Cell length was measured from pole to pole. Time-stamped image capture for relative displacement and speed calculations was performed with Frameshots software (version 3.1.3, EOF Productions, http://www.frame-shots.com/) or ImageJ then imported into Excel and measured with an imported ruler calibrated to the scale bar on each image. Speed was calculated by dividing the displacement by the elapsed time [45]. This measurement technique enabled greater image magnification and was validated by comparison to displacement measurements in both ImageJ and Tracker (version 4.11.0, http://www.cabrillo.edu/~dbrown/tracker/), an Open Source Physics tool (https://physlets.org/tracker/). To compare the velocity, rotation, and displacement of platelet-interacting versus non-interacting treponemes, AVIs were imported into Tracker with frame by frame manual image position correction. Velocity and displacement were calculated as a function in Tracker, and full peak rotations were manually counted. Treponeme rotation was calculated only when an individual peak remained in focus and was averaged per treponeme (mean = 160.90 ± 14.59 frames/ video). Video contrast, brightness, and/or sharpness was enhanced, and video format was change from AVIs to MP4s with PowerPoint 2016 (version 1806, Microsoft, Redmond, WA).

### Flow cytometry

All treatments occurred in microaerophilic conditions with treponemes in tightly closed sterile conical (Sarstedt) or BD Falcon 5 mL polystyrene tubes (BD Canada) when removed from the microaerophilic chamber. For platelet binding assays, viable treponemes were stained with 10 μM carboxyfluorescein succinimidyl ester (CFSE, AAT Bioquest, Sunnyvale, CA) for 30 minutes at room temperature in the dark with gentle rocking. Treponemes were then quenched with one to two volumes of PPP, divided in two, and half the volume was then heat-treated [46,47] (heat-treated treponemes retain CFSE staining). Treponemes and platelets were combined at a ratio of 3:5 treponemes per platelet in PPP at room temperature in the microaerophilic chamber in 5 mL polystyrene tubes (BD Canada), tightly sealed and incubated in the dark at 37˚C. Platelets were pre-activated with 0.1 U/mL bovine thrombin (Sigma-Aldrich, Oakville, ON) for two minutes prior to use. Following 19–24 hours of co- incubation, sample tubes were returned to 5% oxygen and the mixture was incubated with a 1/20 dilution of platelet-specific mouse anti-human CD41a (αIIb integrin) monoclonal antibodies labeled with either PE or PE/Cy5 (clone HIP8, BioLegend, San Diego, CA). After 45 minutes at room temperature, samples were fixed with one volume of ice-cold 2% paraformaldehyde (PFA) in PBS, stored at 4˚C, then diluted with sterile PBS just prior to flow cytometry. Samples were acquired on a BD Fascalibur (BD Canada) with platelet and treponeme populations co-localizing and identified with BD CellQuest acquisition software (BD Canada). Software was set to log scale for both forward scatter (FSC E-1) and side scatter (SSC), and gating was aided by using the SPHERO Flow Cytometry Size Standard Kit (containing polystyrene beads between 2.0 and 9.96 μm from Spherotech, Inc., Lake Forest, IL). Forward and side scatter gating was used to acquire at least 5000 events, and up to four biological replicates were analyzed per sample type. After initial gating by forward and side scatter, FlowJo V10 (FlowJo, LLC, Ashland, OR) analysis further separated subpopulations by the mean fluorescence intensity (MFI) of both platelet (PE or PE/Cy5) and treponeme (CFSE) markers. The shift of platelet and treponeme events positive for both markers to quadrant 2 was counted as a treponeme-platelet binding event. Heat-treated treponemes with or without platelet co-incubation exhibited an auto fluorescence signal that was gated and removed from all samples. For platelet activation assays, co-incubations were conducted in PPP under microaerophilic conditions at either 34°C or 37°C for 2, 4, 8, 20 or 24 hours at a ratio of 5 treponemes to 1 platelet and samples were stained, fixed and prepared for flow cytometry as above. Unlabeled treponemes and platelets were co-incubated, followed by platelet staining with a 1/20 dilution of mouse anti-human monoclonal anti-CD62P (clone AK4, BioLegend, San Diego, CA) labeled with FITC. At least three biological and technical replicates of each sample type were used per experiment. Forward and side scatter gating was conducted as above, followed by quantification of the relative median fluorescence intensity (MFI) of either the activation marker CD41a (PE) or CD62P (FITC). MFI comparisons included platelets that were resting, pre-activated with either 160 μM SFLLRN peptide (Biomatik Corporation, Cambridge, ON) or 5 μg/mL rat collagen I (R & D Systems, Inc., Minneapolis, MN) or co-incubated with *T*. *pallidum*.

### Plate-based fibrin clot production assay

Fibrin clot production by activated platelets was measured in 96 well tissue culture plates (Thermo Scientific Nunc, Waltham, MA) using a method modified from Vinholt *et al* [48]. Control wells included 150 μL of resting PRP + 50 μL of NS/10% NRS. To prepare control activated platelets, either 0.5 U/mL bovine thrombin or 5 μg/mL rat collagen I prepared in NS/10%NRS was added to PRP. Test wells included 50 μL of *T*. *pallidum* (~4.0 x 10^7^ treponemes/mL) in NS/10% NRS added to 150 μL of resting PRP. Plates were incubated at 34°C for 18 hours under microaerophilic conditions, after which the absorbance at 600 nm was read with a BioTek Synergy HT plate reader (Fisher Scientific, Waltham, MA), Results were analyzed using Excel.

### Statistics

A two-way ANOVA analysis evaluated the overall statistical significance of three independent binding experiments assessed by flow cytometry with results shown as means ± the standard deviation (SD). The statistical significance of three independent binding experiments assessed by darkfield microscopy was calculated using an unpaired, two-tailed Student’s t-test and shown as mean ± SD. A Student’s two-tailed t-test was used to assess statistical significance of differences in *T*. *pallidum* morphology, kinematics and levels of platelet activation in individual experiments and is shown as mean ± standard error of the mean (SEM). Statistics were performed, and graphs were constructed, using GraphPad Prism (version 7.04) (GraphPad Software, La Jolla California, USA).

## Results

### *Treponema pallidum* interacts with human platelets

**Fig 1:**
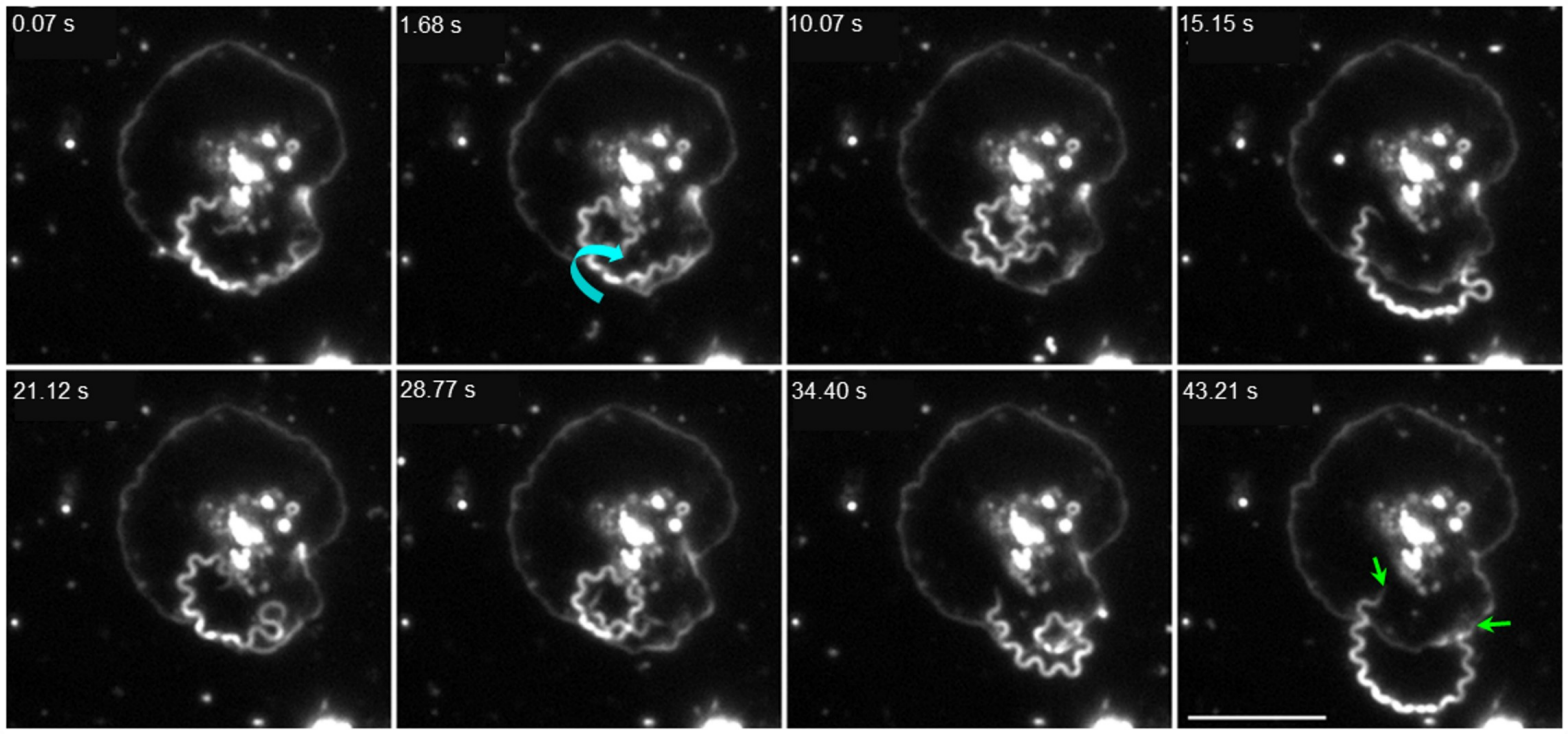
*Treponema pallidum* exhibits a dynamic interaction with human platelets. Darkfield video microscopy panels at 1000x show a treponeme attached to a fully activated platelet by both tips (green arrows 43.21 s). This treponeme alternated between coiling with vigorous axial rotation (2.68 Hz) against the platelet membrane (cyan curved arrow indicates the direction of rotation) (1.68–10.07 s, 28.77 s) and extending above the platelet (15.15 s, 21.12 s, 34.40 s), then remained attached by both tips (43.21 s) for a further 18 min 23 s of observation. Scale bar = 10 μm.

Numerous bacterial species are recognized to interact with human platelets during infection, including the spirochetes *Borrelia burgdorferi* and *Borrelia hermsii* [24,48,49], but to date this potential host cell interaction has remained unexplored for *T*. *pallidum*. To identify potential interactions, human platelets and viable treponemes were co-incubated and observed by darkfield microscopy. High resolution video imaging demonstrated that treponemes interacted with platelets primarily with tip-mediated contact (Fig 1 green arrows). This analysis also revealed interactions to be dynamic with frequent coiling and rotation against (Fig 1 cyan curved arrow) or gliding across the platelet (S1 Video).

### *Treponema pallidum*-platelet interactions correlate with *T*. *pallidum* viability

**Fig 2:**
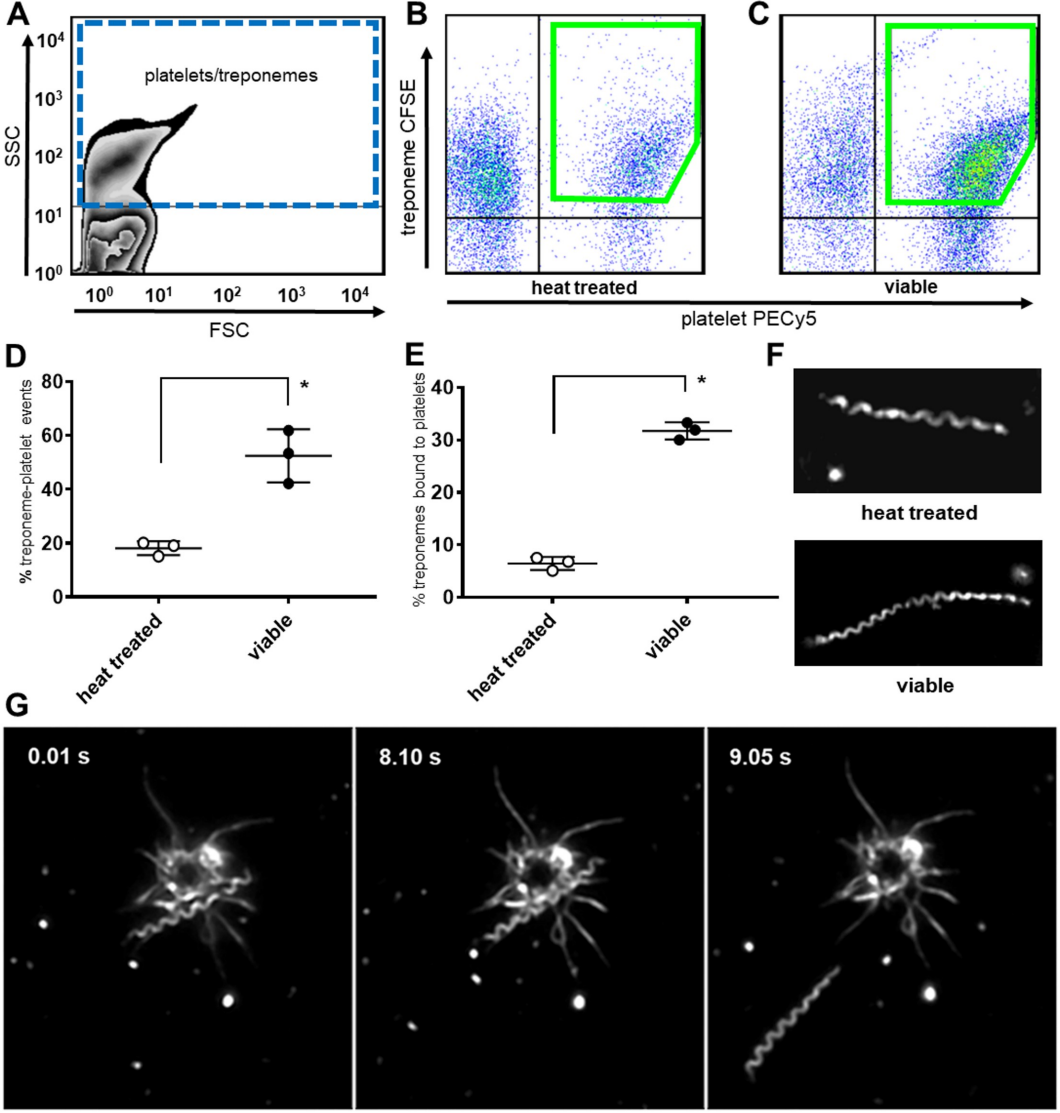
*Treponema pallidum*-platelet interactions. (A) Flow cytometry isolates the co-localized platelet and treponeme populations by SSC and FSC gating. Dot plots of (B) CFSE-labeled heat-treated treponemes demonstrate reduced binding to human platelets labeled with PE/Cy5 anti-CD41a compared with (C) CSFE-labeled viable treponemes. (D) CFSE-labeled viable treponemes bound significantly more human platelets (mean = 55.13% ± 2.91 [SD] *P<0.0001) compared with heat-treated treponemes (mean = 19.05% ± 2.29 [SD]) following co-incubation for 16 hours at 37˚C and ~ 5% oxygen. Results represent the mean of three independent experiments with statistical significance computed by two-way ANOVA, with a minimum of 3 replicates per sample type per experiment. (E) Darkfield microscopy FOV counts (20 random locations/slide) demonstrate viable treponemes bind significantly more human platelets (mean = 31.73% ± 1.19 [SD] *P<0.0001) than heat-treated treponemes (mean = 6.47% ± 1.19 [SD]) following co-incubation at 5% oxygen at 34˚C for 48 hours. Results represent the mean of three independent experiments with statistical significance computed by unpaired two-tailed Student’s t test, n = 3. (F) Heat-treated treponemes (top) are non-motile yet morphologically similar to viable treponemes (bottom). (G) Darkfield microscopy at 1000x magnification demonstrates platelet interactions are reversible. Image capture from video micrographs show edgewise attachment of a treponeme to an activated platelet (0.01 s to 8.10 s) which then detaches and moves away (9.05 s).

To determine if *T*. *pallidum*-platelet interactions were dependent upon viable treponemes, we compared the binding of heat-treated (nonviable) treponemes and viable treponemes to human platelets by flow cytometry (Fig 2A–2D). Platelets and treponemes co-localize and this population formed the first gate on the FSC by SSC plot (blue broken line Fig 2A). The binding events (lime gates) of platelets with either heat-treated (Fig 2B) or viable (Fig 2C) treponemes were compared. Viable treponeme-platelet binding events (mean = 55.13% ± 2.91 [SD] P<0.0001, Fig 2C and 2D) were significantly higher than events associated with heat-treated treponemes (mean = 19.05% ± 2.29 [SD], Fig 2B and 2D). Microscopic analysis consisted of determining the percent of platelet-interacting treponemes out of the total treponemes observed in 20 random locations per slide and confirmed viable treponemes co-incubated with human platelets bound significantly more platelets (mean = 31.73% ± 0.96 [SD] P<0.0001, Fig 2E) compared with heat-treated treponemes (mean = 6.47% ± 0.71 [SD], Fig 2E). Viable treponemes were also observed to disengage from interactions with platelets (Fig 2G panels 0.01, 8.10 & 9.05 s, S3 Fig and S2 Video). Heat treatment resulted in non-motile treponemes (Fig 2F top) that remained morphologically consistent with viable treponemes (Fig 2F bottom).

### *Treponema pallidum*–platelet binding positively correlates with increasing platelet activation

**Fig 3:**
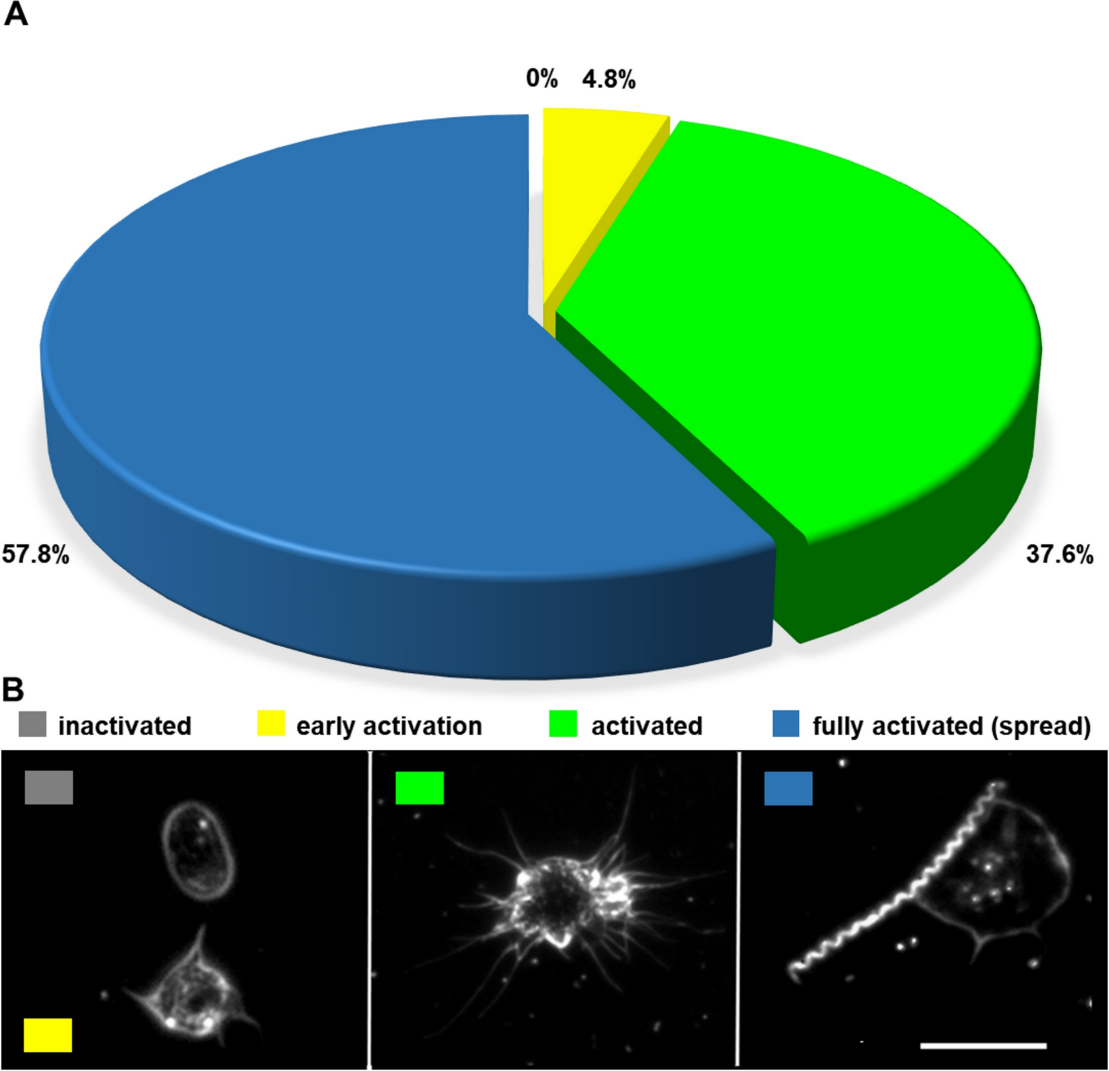
Binding of *T*. *pallidum* to platelets increases with platelet activation. (A) Treponemes displayed preferential binding to fully activated spread platelets (blue), with 57.8% of viewed images showing an interaction between fully activated platelets and *T*. *pallidum*. Treponemes bound to activated spheroid platelets (lime) in 37.6% of images, while platelets in early activation (yellow) constituted only 4.8% of the observed interactions. *Treponema pallidum* was never observed to bind to inactive platelets in 125 interactions analyzed from 75 images containing a total of 422 platelets (S1 Table). Interactions and activation states were observed at 1000x magnification using darkfield microscopy. (B) Images of platelets at different stages of activation at 1000x magnification using darkfield microscopy. Inactive platelets circulate as disks (grey) and bud pseudopods during early activation (yellow). Activated platelets may be spheroid with extended pseudopods (lime) or fully activated with an enlarged surface area and a very thin spread form (blue).

We hypothesized that fully activated, spread platelets would be preferentially targeted by treponemes given the direct adhesion of spread platelets *in vivo* to the endothelium during hemostasis and modulation of vascular permeability [50]. Indeed, when the collection of darkfield images was analyzed there were 125 treponeme-platelet interactions (75 individual images sampled from co-incubations of platelets with 11 independent treponeme extractions), and the majority of treponeme-platelet interactions (57.8%, n = 72) occurred between fully activated, spread platelets (Fig 3A and 3B, right panel, S3 Video) followed by 37.6% to the next most activated state, the spheroid form with fully extended pseudopods (n = 47) (Fig 3A and 3B [middle panel], S3 Video). Platelets in the earliest visible stage of activation, with at least one pseudopod bud (Fig 3A and 3B [left panel], yellow), were observed to interact with treponemes to a much lesser degree at 4.8% (n = 6), whilst at no time were fully inactive, discoid platelets (Fig 3B left panel, grey) observed to interact with *T*. *pallidum*. The preferential interaction between treponemes and spread, activated platelets regularly occurred adjacent to platelets in lesser states of activation in the same FOV, confirming specificity (S1 and S2 Figs). The relative binding of treponemes to pre-activated platelets was close to double that observed for resting platelets (mean = 1.98 ± 0.08 [SEM] P = 0.0003; S5 Fig).

### The interaction of *T*. *pallidum* with platelets is dynamic

**Fig 4:**
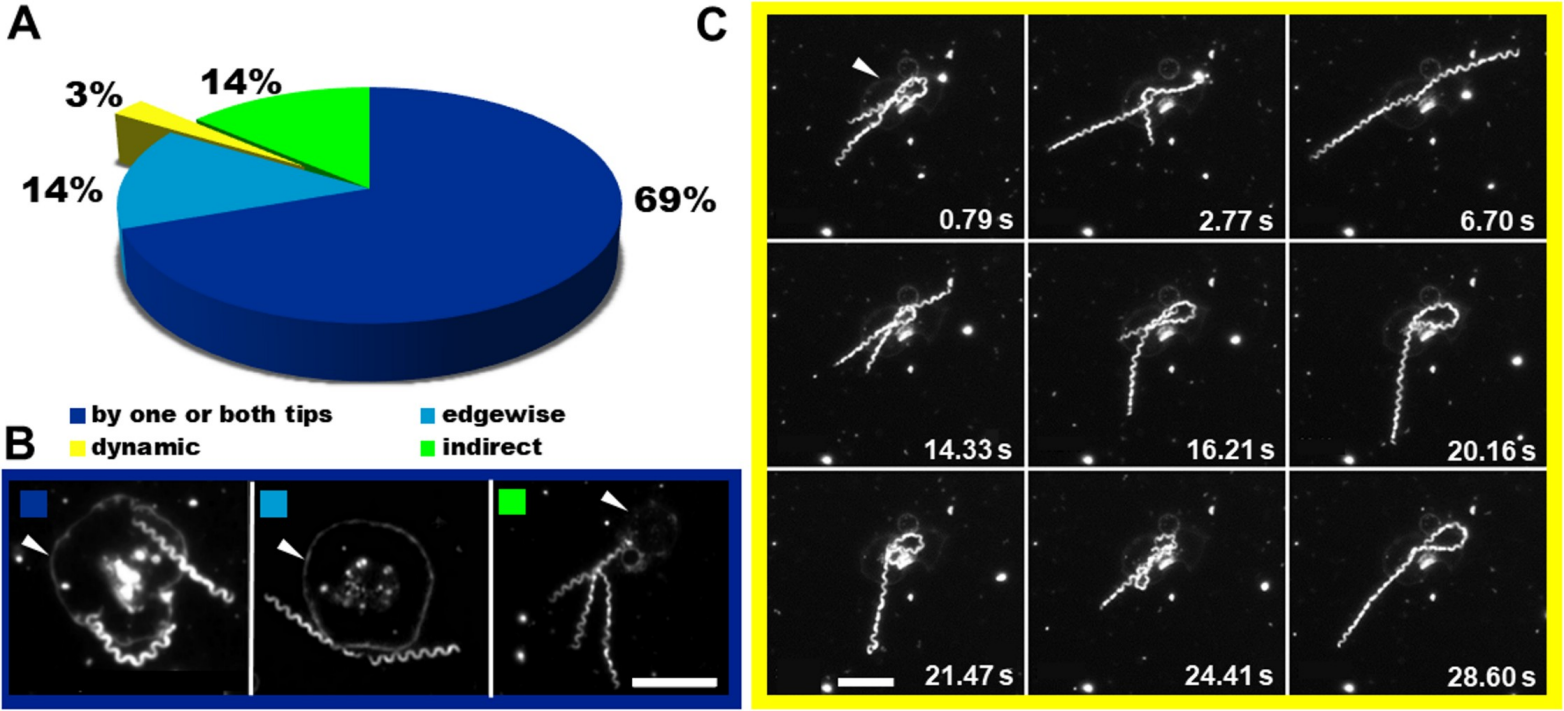
Treponemes bind in multiple ways to activated platelets. (A) Image analysis of 227 interactions in 158 micrographs demonstrated that 69% of treponemes bind platelets using one or both tips (dark blue). Both edgewise binding along the axial plane of the cell body (medium blue) and indirect binding via a platelet-bound treponeme or platelet pseudopod (lime) occurred in 14% of interactions, followed by “dynamic” binding (yellow), which occurred in 3% of interactions. (B) Treponemes interact with one or both tips (left), edgewise (middle) or indirectly via another treponeme (right). (C) Image capture of two treponemes with an activated platelet. Coiling of the treponeme on the platelet (0.79, 14.33, 16.21, 20.16, 21.47 and 24.41 s) with intervening periods of translation and extension away from the platelet by one (2.77, 20.16, 21.47 and 28.60 s) or both (6.70 s) treponemes was observed. White arrowheads indicate fully activated platelets. Scale bars = 5 μm.

Darkfield and electron microscopy studies have previously demonstrated that 90% of *T*. *pallidum* cells adhere to cultured rabbit epithelial by one or both tips, with over a third of treponemes using both tips [51]. Initial darkfield observations revealed a complexity to treponeme-platelet interactions beyond simple adhesion. Over the course of platelet co-incubations with 11 independent treponeme extractions we assembled a collection of live, high resolution images. We categorized and quantified four major types of interactions (227 interactions in 158 micrographs); (1) adhesion by one or both tips (Fig 4A and 4B [left panel], S3 and S4 Videos) occurring at a rate of 69% (n = 227); (2) “edgewise” binding along the axial plane of the cell body (Fig 4A and 4B [middle panel], S4 Video) occurring at a rate of 14%; (3) “indirect” binding by adhesion to a platelet-interacting treponeme or extended platelet pseudopod (Fig 4A and 4B [right panel], S4 Video) occurring at a rate of 14% and; (4) “dynamic” binding, characterized by coiling and uncoiling against the platelet membrane and/or rapid back and forth translations, (Fig 4A and 4C, S4 Video) comprised approximately 3% of interactions. Binding by both tips was observed in less than 1% of *T*. *pallidum*-platelet interactions (S4 Video). “Dynamic” binding involved continuous contact with the platelet but rather than “tip” or “edgewise”adhesion, the treponeme remained in constant motion (Fig 4C, S4 Video).

### *Treponema pallidum* increases rotation and translational velocity upon interacting with platelets

**Fig 5:**
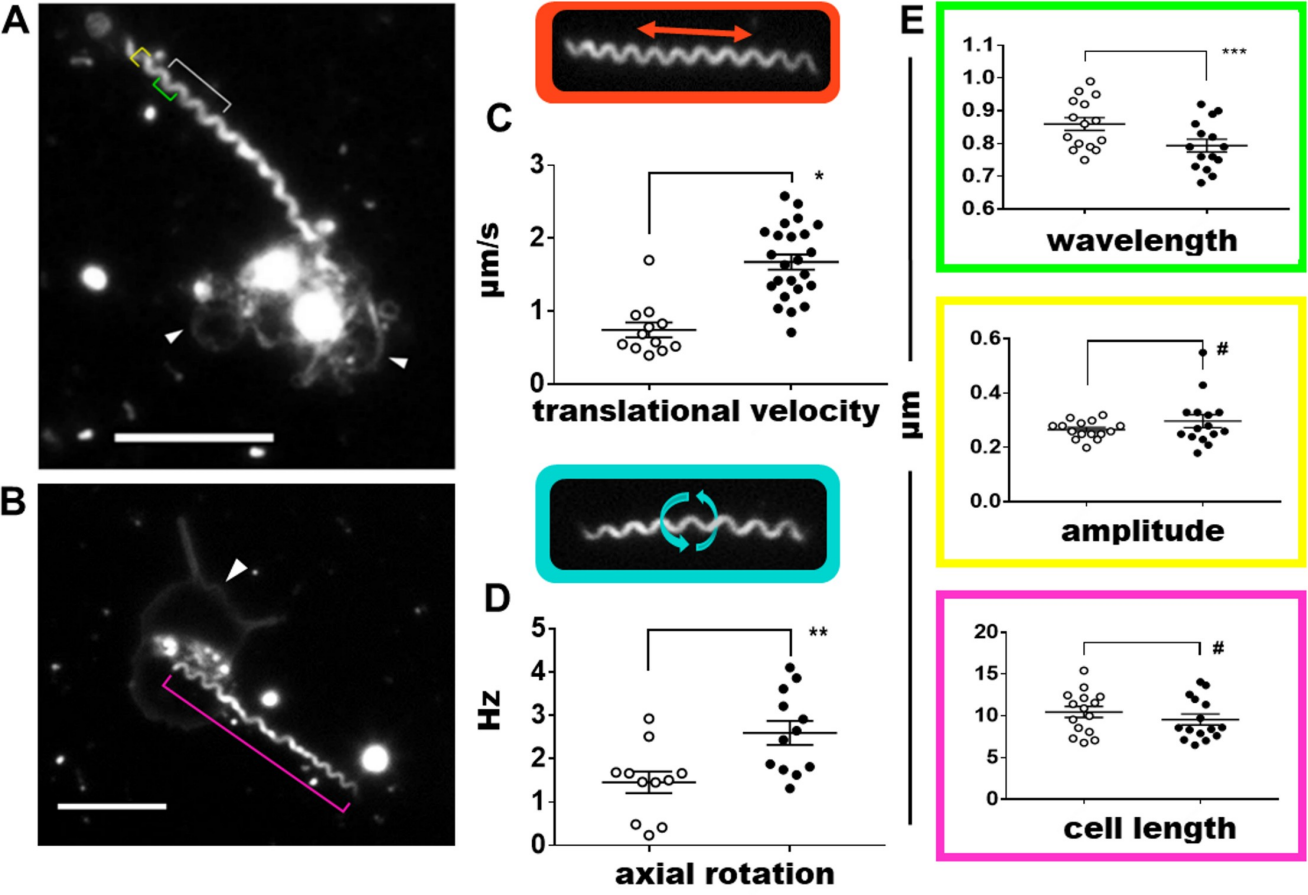
Motility parameters associated with *Treponema pallidum*-platelet interactions. The movement and cell characteristics were contrasted between platelet-interacting (shaded circles) and non-interacting (open circles) treponemes utilizing high-resolution darkfield videos at 1000x magnification. (A) Treponeme wavelength (green bracket) and amplitude (yellow bracket) were measured only when the cell body waveform was in the plane of focus (grey bracket). Two activated platelets (white arrowheads) are bound by one treponeme. The grey bracket shows a segment of the treponeme with cell peak and troughs in the same focal plane. (B) Treponeme length was measured when the full treponeme was in the same focal plane (magenta bracket). (C) Platelet-interacting treponemes demonstrated a higher translational velocity (mean = 1.68 ± 0.10 μm/sec [SEM] *P < 0.0001, n = 24) compared with non-interacting treponemes (mean = 0.65 ± 0.10 μm/sec [SEM], n = 12). (D) Frame by frame analysis determined platelet-interacting treponemes rotate at a higher frequency (mean = 2.60 ± 0.27 Hz [SEM] **P = 0.0060, n = 12) versus non-interacting treponemes (mean = 1.46 ± 0.25 Hz [SEM], n = 11). (E) Top panel: Platelet-interacting treponemes maintained a more tightly coiled helix resulting in a shorter wavelength (mean = 0.79 ± 0.02 μm [SEM] ***P = 0.0237) versus non-interacting treponemes (mean = 0.86 ± 0.02 μm [SEM]), n = 15. Middle panel: Platelet-interacting treponemes demonstrated a trend towards a higher amplitude (mean = 0.30 ± 0.02 μm [SEM]) compared with non-interacting treponemes (mean = 0.27 ± 0.01 μm [SEM]), n = 15. Bottom panel: Platelet-interacting treponemes also displayed a trend towards a shorter, more compact coiled shape (mean cell length = 9.56 ± 0.65 μm [SEM]) compared with non-interacting treponemes (mean cell length = 10.46 ± 0.66 μm [SEM]), n = 15. Student’s unpaired two-tailed t tests were used to calculate statistical significance. Scale bars = 5 μm.

The ability to observe the interaction of treponemes and platelets at high resolution prompted us to compare the motility characteristics of platelet-interacting and non-interacting treponemes in the same FOV. By performing frame-by-frame kinematic comparisons, we detected a significant increase in both the translational velocity (forward and backward motility) and axial rotation of treponemes when engaging with platelets (Fig 5A–5D, S5 Video shows axial rotation of both a platelet-interacting and a non-interacting treponeme). The translational velocity of platelet-interacting treponemes (mean = 1.68 ± 0.10 μm/s [SEM] P < 0.0001) was observed to be over two-fold that of non-interacting treponemes (mean = 0.65 ± 0.01 μm/s [SEM]), with a maximum observed velocity of 2.58 μm/s (Fig 5C). Accompanying this, the rotation rate increased (mean = 2.47 ± 0.86 hertz [SEM] **P = 0.0060) in contrast with non-interacting treponemes (mean = 1.58 ± 0.77 hertz [SEM]) (Fig 5D). Subtle phenotypic changes occurred as the cell body became more compact with a significantly shorter wavelength (ʎ) (mean = 0.79 ± 0.02 μm [SEM] P = 0.0237) compared to that of non-interacting treponemes (mean = 0.86 ± 0.02 μm [SEM]) (Fig 5A green bracket and Fig 5E top). Platelet-interacting treponemes also showed a trend towards a correspondingly higher amplitude (*Α*) (mean = 0.30 ± 0.02 μm [SEM], Fig 5A yellow bracket and Fig 5E middle) and shorter cell body (L) (mean = 9.56 ± 0.65 μm [SEM], Fig 5B magenta bracket and 5E bottom) than non-platelet interacting treponemes (*A* mean = 0.26 ± 0.01 μm [SEM], Fig 5E middle, L mean = 10.46 ± 0.66 μm [SEM], Fig 5E bottom).

### *T*. *pallidum*-platelet interactions reduce treponemal speed and displacement upon plasma movement

**Fig 6:**
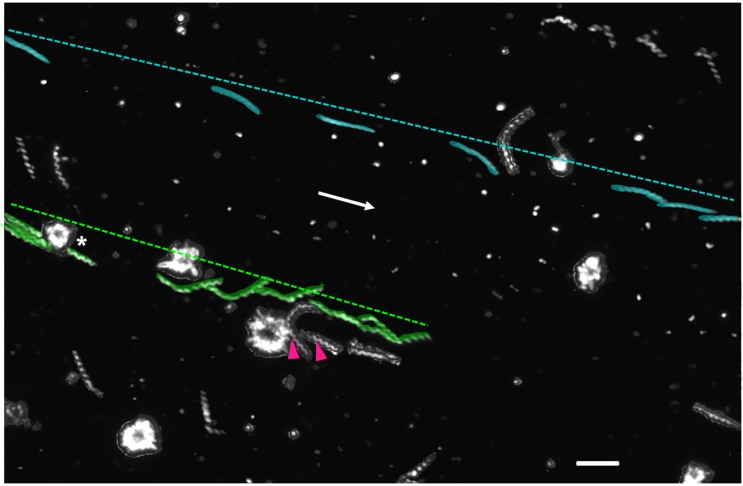
Platelet-tethered treponemes experience reduced displacement and a lower speed than non-interacting treponemes. An overlay of several video microscopy frames demonstrates the reduced speed and displacement experienced by a treponeme (green) that interacted with a slide-adhered platelet (asterisk). This interaction reduced its overall speed to 3.64 μm/sec and displacement to 48.64 μm (green dotted line) compared with a treponeme that did not interact (cyan) which has a speed of 8.73 μm/sec and displacement of 82.98 μm (cyan dotted line). Treponemes engaged in stationary adhesion (magenta arrowheads) maintained attachment when slide movement induced plasma movement. White arrow indicates the direction of flow. Scale bar = 5 μm.

To measure the ability of *T*. *pallidum*-platelet interactions to reduce treponeme displacement, we used high resolution videos to record *T*. *pallidum* movements across the FOV on slides. Prior to reaching equilibrium, plasma currents propelled non-adherent treponemes and platelets across the FOV. Three video image sets that contained both short-term platelet-interacting and non-interacting treponemes were used to compare the effect of platelet adhesion on the ability of treponemes to resist fluid motion until plasma reached a steady state. Treponemes bound to platelets prior to plasma movement maintained attachment, and treponemes that established an interaction with a platelet reduced their speed and overall displacement across the FOV (Fig 6, S3 and S4 Tables). For these measurements the ratio of values for non-interacting to interacting treponemes was compared to determine the differences in relative speed and displacement between different FOV (S3 and S4 Tables). Non-interacting treponemes experienced a higher speed (mean = 2.40 ± 0.16-fold [SEM]) and larger displacement (mean = 2.10 ± 0.30-fold [SEM]) across the FOV compared to platelet-interacting treponemes (S6 Fig, S3 and S4 Tables). The interactions observed in one representative video are shown in Fig 6. By establishing an interaction with a platelet, treponeme 1 (Fig 6 green) reduced its overall speed to 3.64 μm/sec and displacement to 48.64 μm during 13.35 s of viewing. In comparison, treponeme 2 (Fig 6 cyan), which did not interact with platelets as it passed across the FOV, had a speed of 8.73 μm/sec and displacement of 82.98 μm during the 9.51 s it was visible in the FOV (S3 Table, S6 Video).

### *Treponema pallidum* activates human platelets

**Fig 7:**
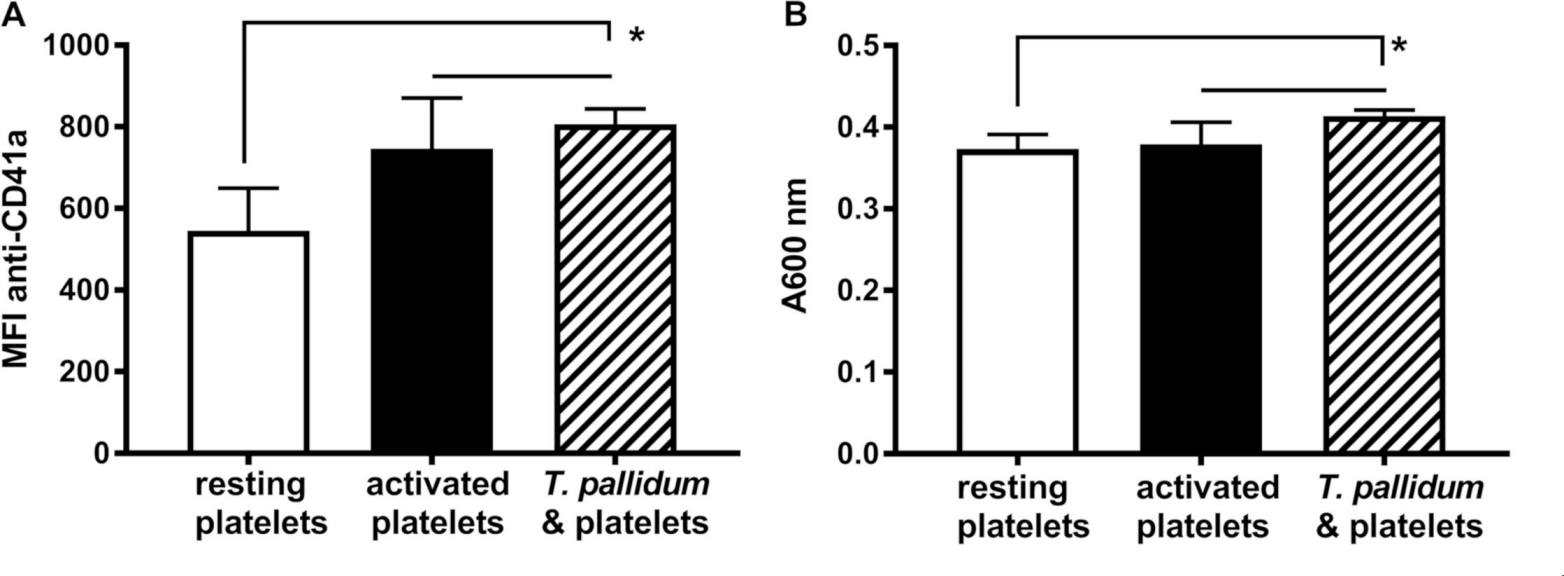
*Treponema pallidum* activates human platelets. The potential for treponemes to activate platelets was assessed by flow cytometry (A) and using a plate-based assay (B). (A) Following 18 hours co-incubation at 37°C the results of two independent flow cytometry assays quantified platelet activation by the median fluorescence intensity (MFI) associated with CD41a receptor up-regulation. Platelets co-incubated with treponemes (hatched bar) demonstrated a significantly higher MFI (mean = 806.0 ± 37.76 [SEM], *P = 0.0118, n = 25) compared to resting platelets (white bar) (mean = 545.5 ± 104.4 [SEM], n = 18). There was no significant difference between treponeme co-incubated platelets and agonist pre-activated platelets (black bar) (mean = 746.5 ± 124.3 [SEM], n = 18). (B) Following co-incubation for 18 hours at 34°C the results of two independent platelet activation experiments were assessed by measuring the absorbance at 600 nm as a surrogate measure of fibrin clot formation. Platelets co-incubated with *T*. *pallidum* (hatched bar) had a higher absorbance (mean = 0.4137 ± 0.007 [SEM], *P = 0.0221, n = 20) compared to resting platelets (white bar) (mean = 0.3730 ± 0.018 [SEM], n = 12). There was no significant difference between treponeme-co-incubated platelets and activated platelets (black bar) (mean = 0.3791 ± 0.027 [SEM], n = 7).

The potential for *T*. *pallidum* to activate platelets was investigated by quantifying platelet receptor CD41a expression by flow cytometry. Co-incubation of *T*. *pallidum* with resting platelets elicited a significant increase in CD41a expression (mean = 806.0 ± 37.76 MFI [SEM], P = 0.0118) compared to resting platelets (mean = 545.5 ± 104.4 MFI [SEM]) (Fig 7A). The level of platelet activation by treponemes was comparable to that seen with agonist-activated platelets (mean = 746.5 ± 124.3 MFI [SEM], Fig 7A).

Platelet activation by *T*. *pallidum* was also assessed by probing for the production of fibrin clots using a plate-based assay to measure absorbance at 600 nm. In this assay, an increase in absorbance is indicative of fibrin clot formation, which occurs as a result of platelet activation. As shown in Fig 7B, incubation of resting platelets with *T*. *pallidum* resulted in an absorbance increase (mean = 0.4137 ± 0.007 [SEM], P = 0.0221) compared to that of resting platelets alone (mean = 0.3730 ± 0.018 [SEM]). As with the flow cytometry experiments, the level of platelet activation by treponemes was comparable to that seen with agonist-activated platelets (mean = 0.3791 ± 0.027 [SEM], Fig 7B).

Upon initial exposure to *T*. *pallidum*, platelets displayed no significant increase in the CD62P MFI (S7A Fig, blue box, mean = 59.69 ± 1.11 [SEM]) compared to inactive platelets (mean = 56.18 ± 0.37 [SEM]). After this 4 hour lag period, treponeme co-incubated platelets expressed significantly more CD62P (mean = 64.77 ± 1.57 [SEM], P = 0.0002) compared with inactive platelets (mean = 53.62 ± 0.72 [SEM]|) and this trend continued with significant results at 8 hours (P < 0.0001), 20 hours (P < 0.001) and 24 hours (P = 0.0013).

A similar lag period was observed in a separate experiment which tested the platelet binding potential of heat-treated versus viable treponemes, with no significant difference in platelet binding after 1 hour co-incubation (blue box S7B Fig) with viable (mean = 23.39 ± 3.92 [SEM]) or heat-treated treponemes (mean = 17.36 ± 0.73 [SEM]). Significantly increased platelet binding was seen after 3 hours (mean = 31.65 ± 4.75 [SEM], P = 0.0342) and 15 hours (mean = 31.12 ± 1.67 [SEM], P = 0.0016) of co-incubation with viable treponemes, compared to the level of binding observed with heat-treated treponemes at 3 hours (mean = 16.44 ± 0.83) and 15 hours (mean = 17.17 ± 0.77 [SEM]).

## Discussion

In this study we have established, for the first time, that live *T*. *pallidum* interacts with human platelets. By using a modified darkfield microscopy method, wherein the slide volume is reduced to enhance resolution, we were able to compare physical and kinematic parameters between platelet-interacting and non-interacting treponemes. Platelet-interacting treponemes increased their axial rotation and translational velocity and decreased their wavelength compared with non-interacting treponemes, to an extent that achieved statistical significance. A trend towards increased amplitude and decreased length was also observed with platelet-interacting treponemes compared to non-interacting treponemes, although these parameters did not achieve statistical significance. Interestingly, *B*. *burgdorferi* has demonstrated decreased cell length following co-incubation with soluble fibronectin, present in plasma at 300–400 μg/mL [52,53].

By comparison with a previous report that investigated the translational velocity, wavelength and amplitude of free *T*. *pallidum* at a 400x magnification, our measured amplitudes were comparable (0.30 ± 0.02 μm and 0.26 ± 0.01 μm compared to 0.28 ± 0.01 μm reported by Harman et al [44]). However, notable differences in translational velocity and wavelength were observed. In our study, the translational velocity of free treponemes in human plasma was slower (mean = 0.65 ± 0.01 μm/sec) compared to the translational velocity previously reported for *T*. *pallidum* in CMRL medium (mean = 1.9 ± 0.2 μm/s) [44]. Similarly, we observed a wavelength of 0.86 ± 0.02 μm for free treponemes while Harman et al reported a wavelength of 1.56 ± 0.04 μm [44]. Possible reasons for these differences include the fact that our study was conducted at a higher magnification, potentially allowing for increased accuracy of measurements, as well as the possibility of altered environmental conditions increasing treponemal health, with our use of a microaerophilic chamber for *T*. *pallidum* extraction and nutrient-rich platelet suspensions. Further, Harman et al reported a reduction of *T*. *pallidum* translational velocity upon increasing viscosity of the treponemal suspension buffer [42]. Since human plasma has a higher viscosity than CMRL medium (1.10–1.60 cP [43,44] compared to 0.89 cP [45], respectively), this may explain the observed difference in translational velocity between the two studies.

The current study shows that *T*. *pallidum* exhibits an altered phenotype upon interacting with human platelets. Overall, we observed that *T*. *pallidum* achieves a more compressed spiral shape and increased activity upon interaction with human platelets, and that *T*. *pallidum* preferentially interacts with spread, activated platelets. Further, we observed that *T*. *pallidum-*platelet interactions occurred only with viable (and not heat-killed) treponemes, were reversible, and increased the translational velocity of *T*. *pallidum*. Collectively these findings suggest the interaction of *T*. *pallidum* with platelets is an active process that could contribute to treponemal pathogenesis, rather than a platelet-initiated process to aid in treponemal elimination during infection.

Relevantly, the current study suggests *T*. *pallidum* both interacts with activated platelets and activates platelets from an unactivated state over a time period of approximately 4 hours after the time of first interaction. Regardless of the route of activation, the interaction of *T*. *pallidum* with spread, activated platelets could contribute to treponeme persistence and extravasation. Activated platelets adhere to vascular breaches in a manner dependent upon the interaction of the platelet receptor GlycoproteinVI with collagen, facilitating platelet retainment *in situ* for several hours [54,55]; *in vivo* this would avoid the clearance observed with circulating, activated platelets [56–58]. Further, during inflammation endothelial cell engulfment of adherent platelets occurs [59], which could aid in the extravasation of co-adherent *T*. *pallidum*.

The significance of the demonstrated association of *T*. *pallidum* with platelets, and in particular activated platelets, is expected to be two-fold. First, the association may provide *T*. *pallidum* with nutrients which enable enhanced fitness, since activated platelets secrete a vast array of small molecules, ions and enzymes into the surrounding cellular milieu [46,48–50]. Second, the activated platelet secretome has also been demonstrated to facilitate platelet-endothelial cell adhesion and alter vascular permeability [19,47,49–51,60]. Endothelial-bound activated platelets play a key role in mediating *Streptooccus gordonii* and *Streptococcus oralis* adhesion during infective endocartitis [61–63]. Metastatic tumor cells have been shown to directly interact with, and rely on, platelets to faciltiate extravasation [19,21,64], and extravasation and dissemination of *T*. *pallidum* may be similarly aided by *T*. *pallidum*-platelet interactions. In this scenario, the interaction of *T*. *pallidum* with platelets would reduce treponemal speed and limit treponemal displacement within the bloodstream, as suggested by the observations reported in this study, allowing *T*. *pallidum* to be poised for extravasation. The *T*. *pallidum*-platelet interactions would further assist with extravasation via the natural association of activated platelets with endothelial cells and the capacity of the secretome from activated platelets to increase vascular permeability [60,65]. In the current study nearly one third of treponemes bound to platelets in a heterogeneous manner, which differs from the preferential tip binding noted to dominate direct binding of *T*. *pallidum* to endothelial cells from prior studies [40]. When considered in the context of the increased translational velocity observed for platelet-interacting treponemes, this may provide an opportunity for *T*. *pallidum* to navigate platelet-endothelial cell associations and access areas of increased vascular permeability.

In addition to increasing vascular permeability, platelets also increase blood-brain barrier permeability through secretion of sCD40L, VEGF, IL-1, CXCL4/PF4 and serotonin [66–69]. Further, maternal platelets are incorporated into the lumen of blood vessels during placental formation and are found in the placental villi and fetal endothelium during the first trimester of pregnancy [70,71]. The ability of *T*. *pallidum* to interact with platelets may therefore also play a role in the capability of *T*. *pallidum* to cross the blood-brain and placental barriers.

While the molecular details of the observed *T*. *pallidum*-platelet interactions have yet to be elucidated, prior investigations focused on *T*. *pallidum* and other pathogens suggest multiple mechanisms may contribute to this interaction [72–74]. The related spirochetes *B*. *burgdorferi* and *B*. *hermsii* bind directly to the platelet receptor β3 integrin via an outer membrane protein [24,75], and *T*. *pallidum* proteins bind the plasma proteins fibrinogen and [76] fibronectin [77], which in turn bind to the αIIbβ3, αvβ3 and α5β1 integrins found on platelets [72,78]. Platelet activation is both paracrine and autocrine, mediated by granule secretion and synergy between signaling pathways [79]. Activation of platelets via collagen engagement of the GlycoproteinVI platelet receptor induces specific signaling, hemostasis-independent single platelet adhesion to inflamed endothelium [54,55] and ADP secretion, which in turn promotes autocrine activation, high affinity integrin RGD-ligand interactions and platelet spreading [79–81]. The significance of platelet-mediated extravasation to metastasis also suggests an important role for increased platelet sensitivity to ADP during late metastasis [82,83], a feature also reported during syphilis infections [35]. Thus, *T*. *pallidum* may interact with platelets via plasma proteins and platelet receptors using a mechanism common to many bacterial pathogens, and at the same time exploit mechanisms used by metastatic cells to facilitate hematogenous dissemination.

The current study reports the novel finding that *T*. *pallidum* attaches to platelets, with a preference for activated platelets. Determination of the relevance of this association to the process of *T*. *pallidum* pathogenesis will require further investigations that extend beyond observational findings to probe the molecular details, and *in vivo* relevance, of the *T*. *pallidum*-platelet interaction.

## Supporting information

S1 Fig *Treponema pallidum* preferentially binds activated platelets. UVDFM image of platelets in the same FOV in both the inactive state and all stages of activation: inactive (grey arrowhead), early activation (yellow arrowhead), activated spheroid (lime arrowhead) and fully activated spread (blue arrowheads) with both direct and indirectly bound treponemes. Scale bar = 5 μm.
(TIF)

S2 Fig *Treponema pallidum* attached to a fully activated spread platelet. A treponeme is attached by one tip to a fully activated, spread platelet (blue arrowhead) with an activated spheroid platelet adjacent (lime arrowhead). Scale bar = 5 μm.
(TIF)

S3 Fig *Treponema pallidum* adhesion to platelets is a reversible interaction. Frame capture from UVDFM videos demonstrate interactions between two treponemes and a slide-anchored activated platelet (yellow outline). The treponemes engage in dynamic binding with one treponeme leaving the platelet at 4 min 6.5 s and returning to re-engage the platelet at 4 min 10.1 s. Scale bar = 5 μm.
(TIF)

S4 Fig Treponemes and platelets co-localize by size. Treponeme-platelet co-localization is demonstrated in flow cytometry FSC x SSC dot plots showing the position of 1, 2, 5, and 10 micron sized beads (cyan) overlaid with representative dot plots of *T*. *pallidum* only (magenta), platelet only (lime) and *T*. *pallidum*-platelet co-incubation (black) samples.
(TIF)

S5 Fig Pre-activating platelets results in higher treponeme binding. Flow cytometry quantified the binding of CFSE-labeled viable or heat treated treponemes to either resting (defined as resting prior to co-incubation) or pre-activated platelets stained with PE-labeled anti-CD41a (three biological replicates per sample type). The number of viable treponeme (hatched bars)-resting platelet interactions was designated as the baseline (and set at 1.0) and used to compare viable treponeme binding to pre-activated platelets. Platelet pre-activation nearly doubled the binding events (mean = 1.98 ± 0.08 [SEM] ***P = 0.0003). Compared to viable treponemes, heat treated treponemes bound significantly fewer resting platelets (grey bar) (mean = 0.187 ± 0.02 [SEM] ***P = 0.0003) and pre-activated platelets (black bar) (mean = 0.98 ± 0.02 [SEM] P = 0.0003).
(TIF)

S6 Fig Platelet interactions reduce the relative velocity and relative displacement of platelet-interacting treponemes. The ratio of non-interacting treponemes to platelet-interacting treponemes was utilized to normalize the values for relative velocity and displacement of treponemes between three different videos. Treponemes that did not engage platelets experienced a greater than two-fold relative velocity (grey bars) and displacement (red line) increase compared to treponemes engaged in platelet tethering.
(TIF)

S7 Fig Treponemes bind to and activate platelets after a lag period. Two time course experiments demonstrate a lag period (blue boxes) for platelet activation (A) and maximal binding (B). (A) The MFI associated with the expression level of platelet activation marker CD62P was compared between initially inactive platelets (white bars) and those co-incubated with viable *T*. *pallidum* (hatched bars) after 2, 4, 8, 20 and 24 hours at 37°C. After 2 hours (blue box) there was no significant increase in CD62P up-regulation between inactive platelets (mean = 56.18 ± 0.37 [SEM], n = 4) and treponeme co-incubated platelets (mean = 59.69 ± 1.11 [SEM], n = 14). Significant CD62P expression was seen in platelets co-incubated with treponemes after 4 hours (mean = 64.77 ± 1.57 [SEM], ***P = 0.0002, n = 7), 8 hours (mean = 60.84 ± 0.95 [SEM], ****P < 0.0001, n = 5), 20 hours (mean = 64.45 ± 1.20 [SEM], ****P < 0.0001, n = 5) and 24 hours (mean = 64.23 ± 1.56 [SEM], **P = 0.0013, n = 4) compared to inactive platelets at 4 hours (mean = 53.62 ± 0.72 [SEM], n = 5), 8 hours (mean = 51.0 ± 0.42 [SEM], n = 5), 20 hours (mean = 53.98 ± 0.83 [SEM], n = 5) and 24 hours (mean = 56.56 ± 0.48 [SEM], n = 5). (B) Platelet binding was compared by flow cytometry for CFSE-labeled viable (black circles) or CSFE-labeled heat-treated treponemes (open circles) to platelets stained with PE-labeled antiCD41a after 1, 3, or 15 hours co-incubation at 37°C. After 1 hour co-incubation (blue box) there was no significant difference in platelet binding between viable (mean = 23.39 ± 3.92 [SEM], n = 3) and heat-treated (mean = 17.36 ± 0.73 [SEM], n = 3) treponemes. Viable treponemes bound significantly more platelets after 3 hours (mean = 31.65 ± 4.75 [SEM], *P = 0.0342, n = 3) and 15 hours (mean = 31.12 ± 1.67 [SEM], **P = 0.0016, n = 3) compared to heat-treated treponemes after 3 hours (mean = 16.44 ± 0.83 [SEM], n = 3) or 15 hours (mean = 17.17 ± 0.76 [SEM], n = 3).
(TIF)

S1 Table Treponeme interactions with platelets increases with platelet activation state. (DOCX)

S2 Table A comparison of the physical and kinematic parameters of platelet-interacting and non-interacting treponemes. (DOCX)

S3 Table Relative velocity and displacement of platelet-interacting and non-interacting treponemes in three representative videos. (DOCX)

S4 Table Ratios of non-interacting to platelet-interacting treponemes compares relative velocity and relative displacement. (DOCX)

S1 Video A treponeme interacts with a spread platelet in a complex manner. (MP4)

S2 Video Treponeme-platelet interactions are reversible. (MP4)

S3 Video Treponemes interact with activated platelets. (MP4)

S4 Video Treponemes bindi to activated platelets; by both tips, edgewise interactions, edgewise binding by actively rotating ring-shaped treponemes, indirectly via another treponeme, and by dynamic interactions. During dynamic interactions treponemes move vigorously across an activated platelet.
(MP4)

S5 Video Platelet-interacting treponemes rapidly rotate. A platelet-interacting treponeme approaching cell division rotates rapidly on a spread platelet with a non-interacting treponeme rotating more slowly (bottom of frame).
(MP4)

S6 Video Platelet tethering reduces treponeme relative velocity and relative displacement. (MP4)
