# Supplementary figures and images for: Interaction of Treponema pallidum, the syphilis spirochete, with human platelets
Source: PLoS One. 2019 Jan 18;14(1):e0210902. doi: 10.1371/journal.pone.0210902 (PMC6338379; doi:10.1371/journal.pone.0210902)

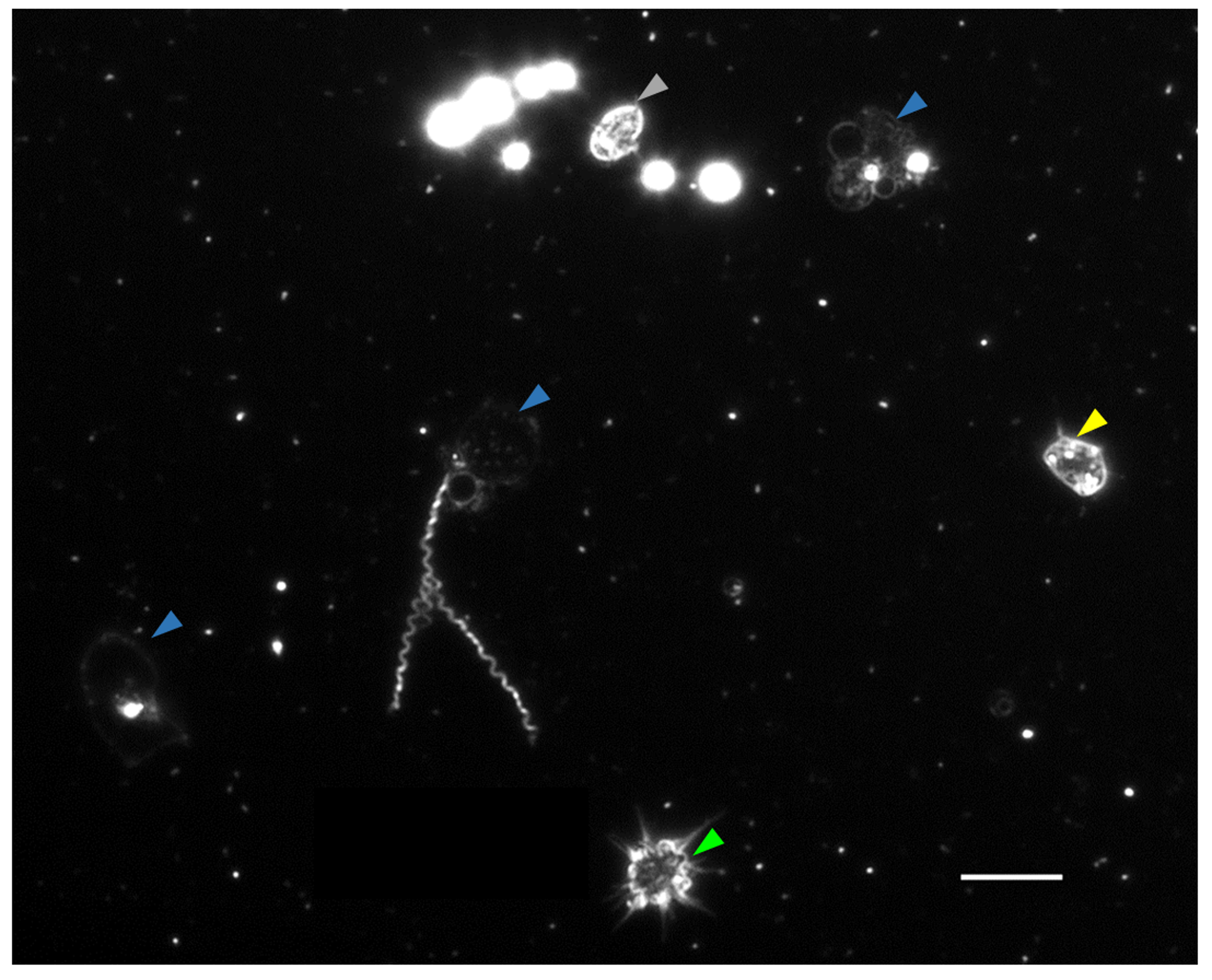

Supplement: S1 Fig — UVDFM image of platelets in the same FOV in both the inactive state and all stages of activation: inactive (grey arrowhead), early activation (yellow arrowhead), activated spheroid (lime arrowhead) and fully activated spread (blue arrowheads) with both direct and indirectly bound treponemes. Scale bar = 5 μm. (TIF) [file pone.0210902.s001.tif]

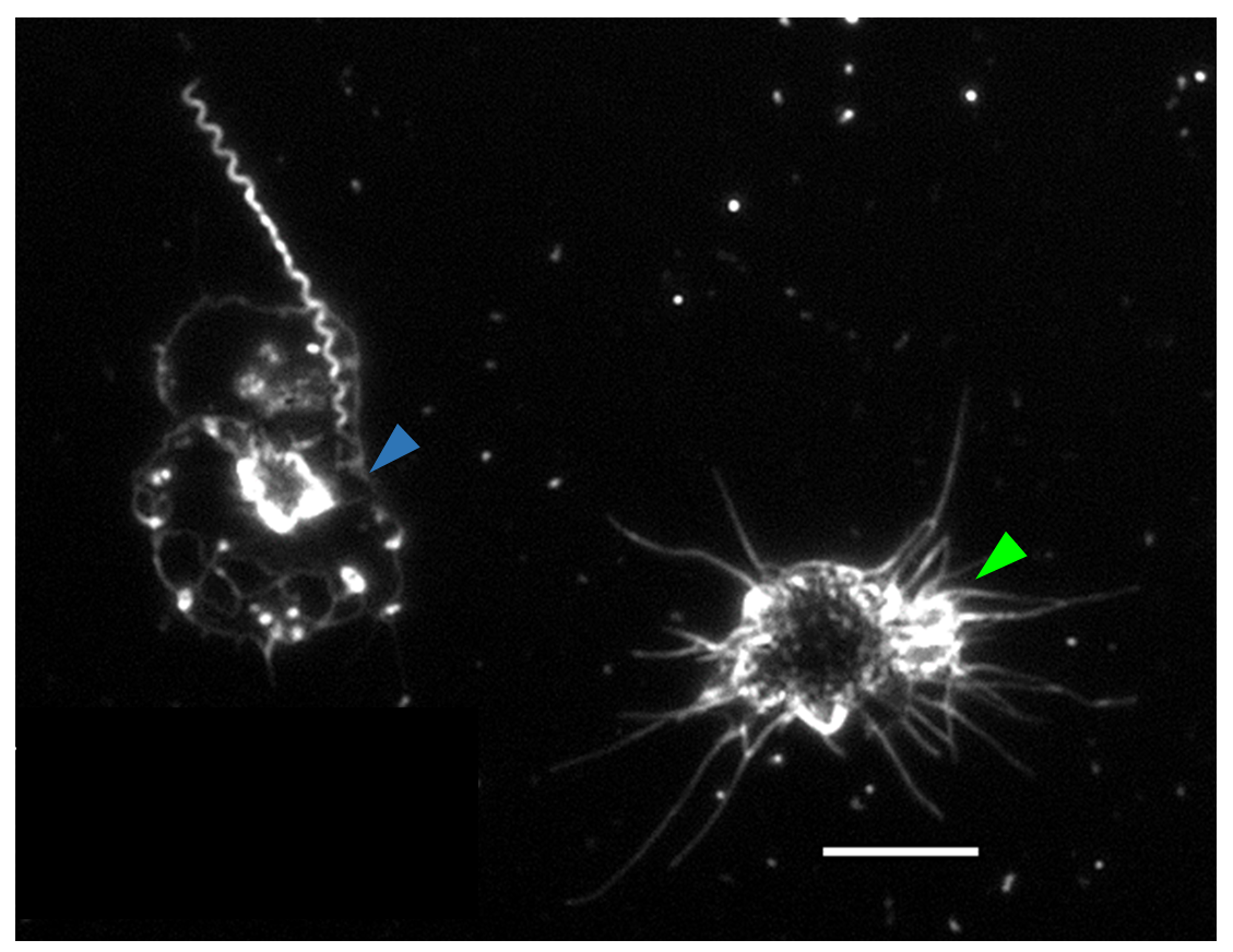

Supplement: S2 Fig — A treponeme is attached by one tip to a fully activated, spread platelet (blue arrowhead) with an activated spheroid platelet adjacent (lime arrowhead). Scale bar = 5 μm. (TIF) [file pone.0210902.s002.tif]

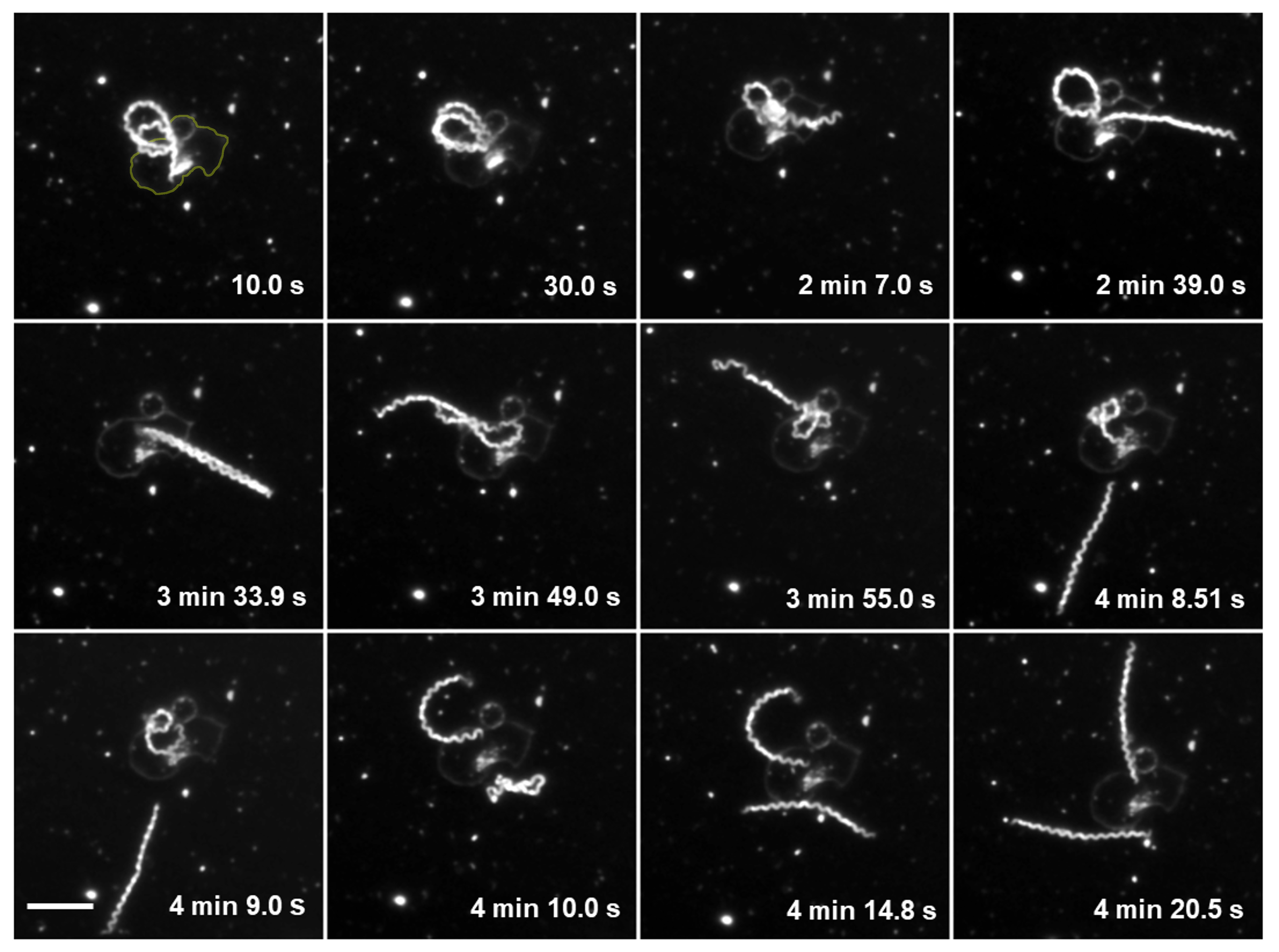

Supplement: S3 Fig — Frame capture from UVDFM videos demonstrate interactions between two treponemes and a slide-anchored activated platelet (yellow outline). The treponemes engage in dynamic binding with one treponeme leaving the platelet at 4 min 6.5 s and returning to re-engage the platelet at 4 min 10.1 s. Scale bar = 5 μm. (TIF) [file pone.0210902.s003.tif]

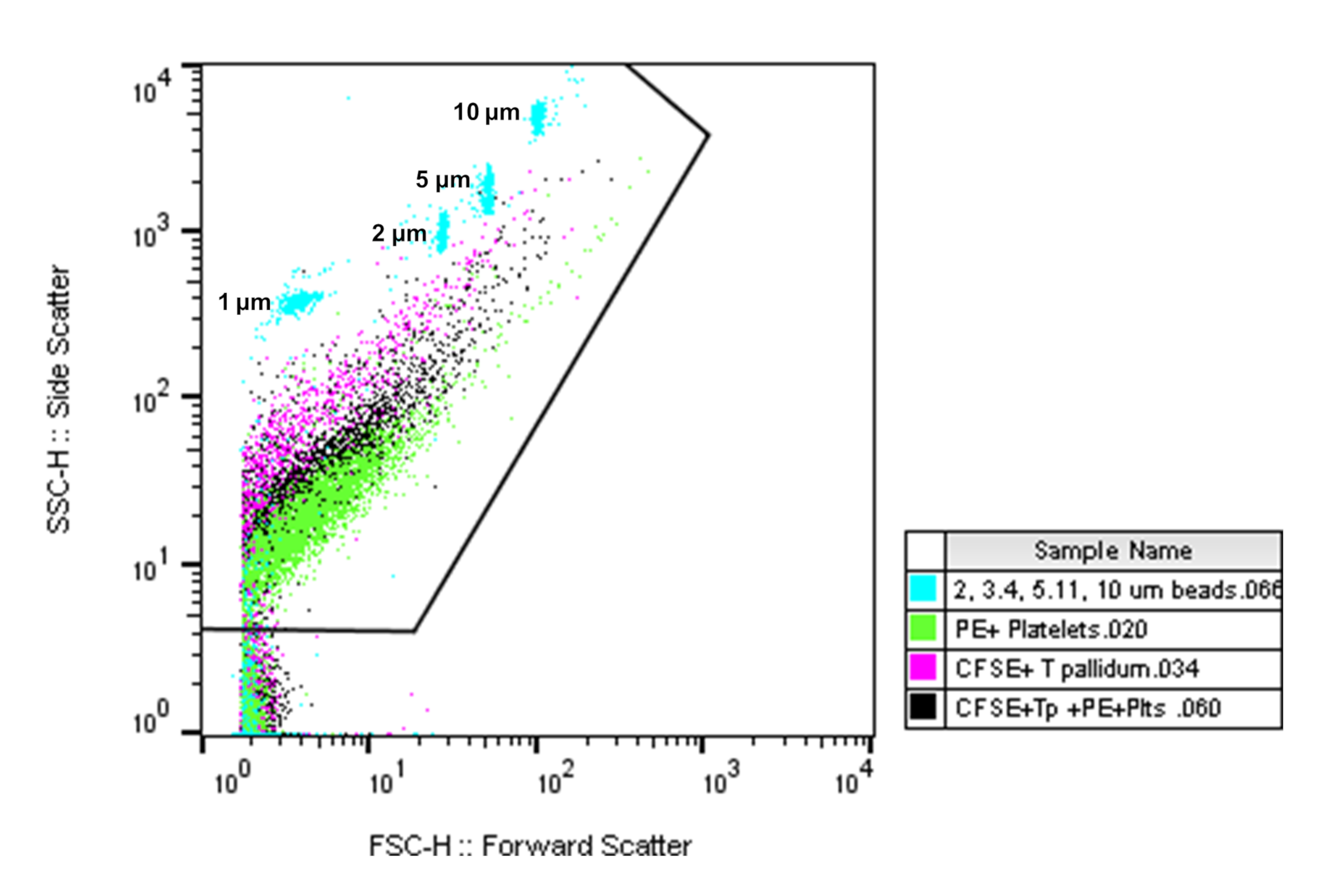

Supplement: S4 Fig — Treponeme-platelet co-localization is demonstrated in flow cytometry FSC x SSC dot plots showing the position of 1, 2, 5, and 10 micron sized beads (cyan) overlaid with representative dot plots of T. pallidum only (magenta), platelet only (lime) and T. pallidum-platelet co-incubation (black) samples. (TIF) [file pone.0210902.s004.tif]

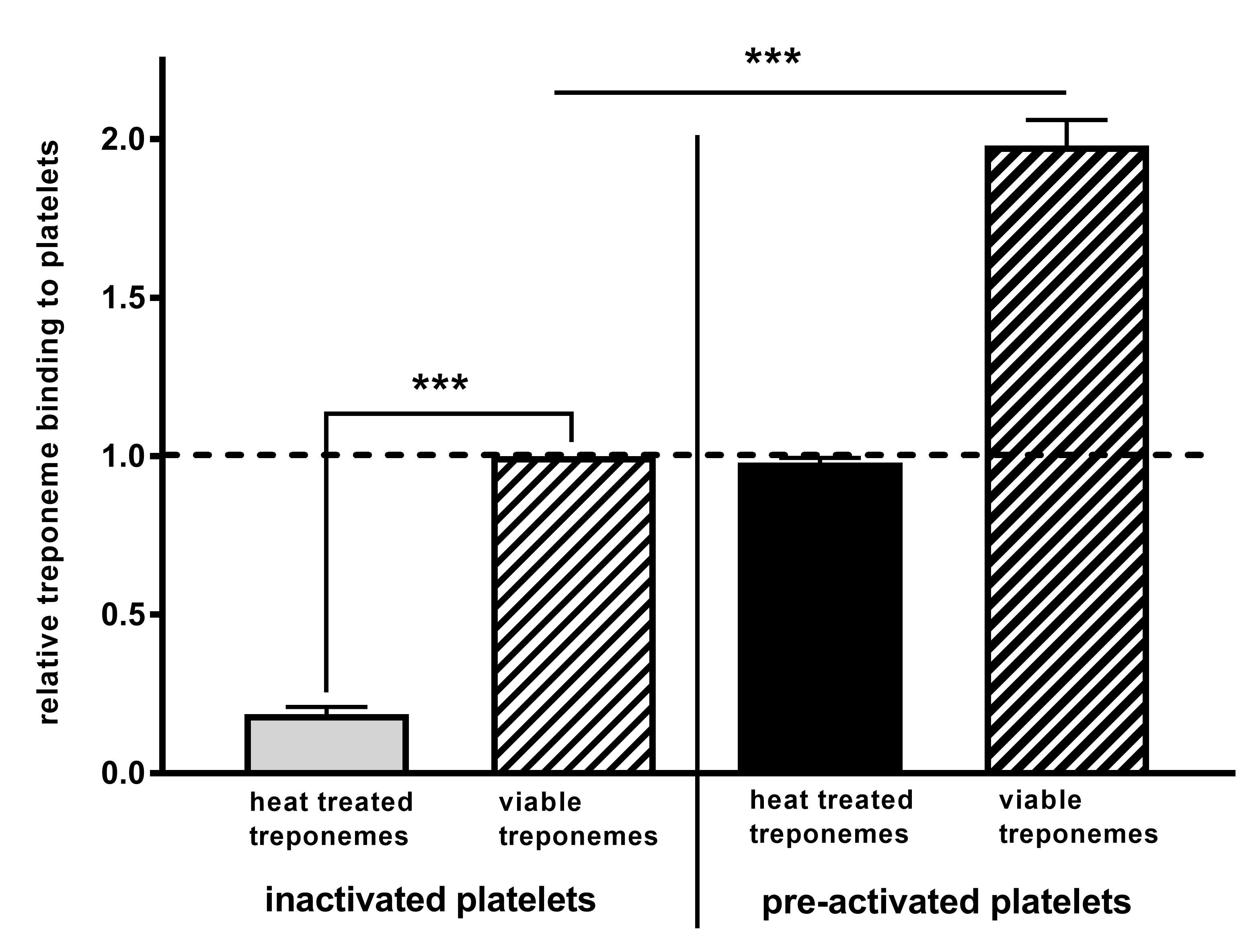

Supplement: S5 Fig — Flow cytometry quantified the binding of CFSE-labeled viable or heat treated treponemes to either resting (defined as resting prior to co-incubation) or pre-activated platelets stained with PE-labeled anti-CD41a (three biological replicates per sample type). The number of viable treponeme (hatched bars)-resting platelet interactions was designated as the baseline (and set at 1.0) and used to compare viable treponeme binding to pre-activated platelets. Platelet pre-activation nearly doubled the binding events (mean = 1.98 ± 0.08 [SEM] ***P = 0.0003). Compared to viable treponemes, heat treated treponemes bound significantly fewer resting platelets (grey bar) (mean = 0.187 ± 0.02 [SEM] ***P = 0.0003) and pre-activated platelets (black bar) (mean = 0.98 ± 0.02 [SEM] P = 0.0003). (TIF) [file pone.0210902.s005.tif]

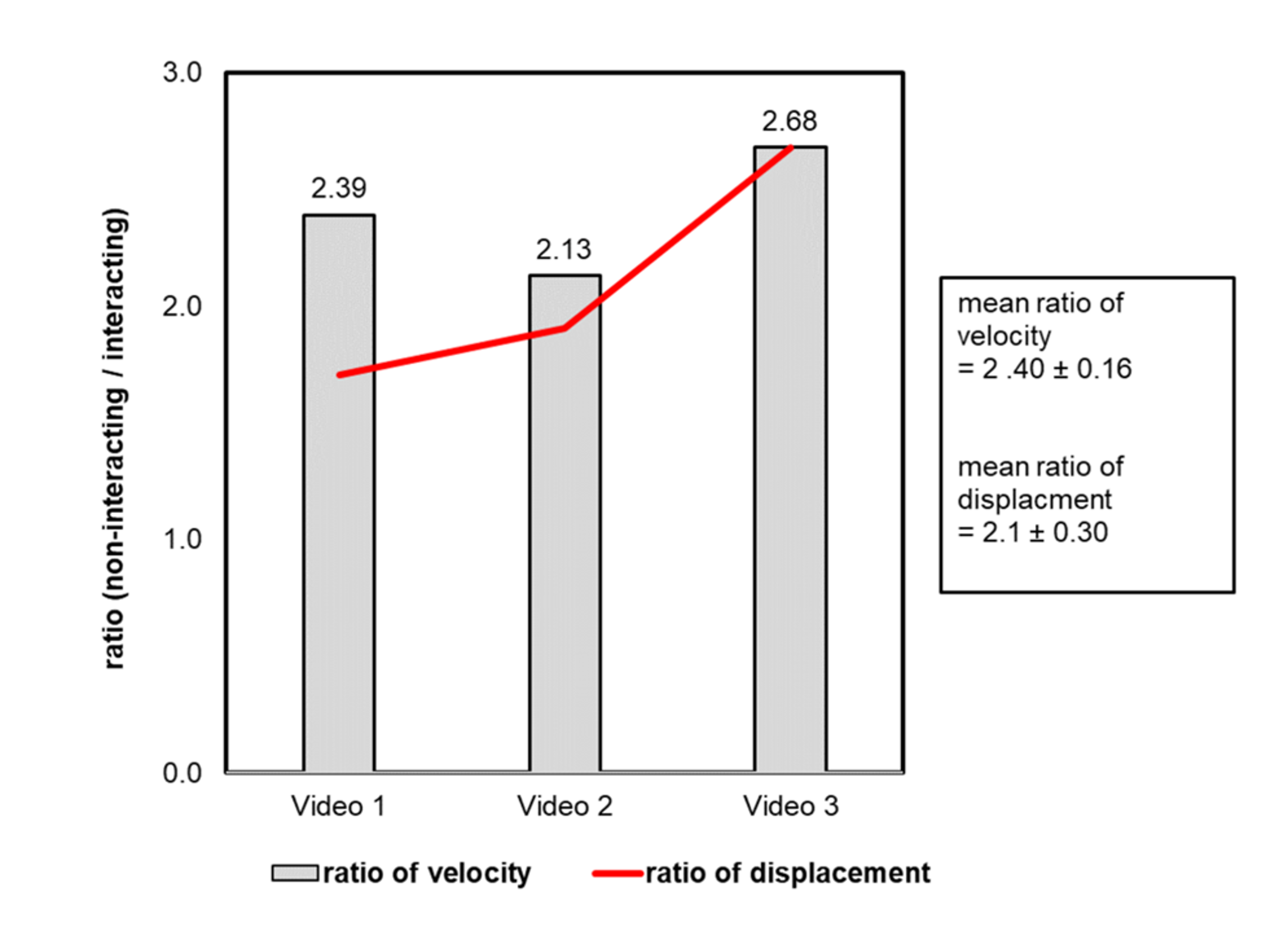

Supplement: S6 Fig — The ratio of non-interacting treponemes to platelet-interacting treponemes was utilized to normalize the values for relative velocity and displacement of treponemes between three different videos. Treponemes that did not engage platelets experienced a greater than two-fold relative velocity (grey bars) and displacement (red line) increase compared to treponemes engaged in platelet tethering. (TIF) [file pone.0210902.s006.tif]

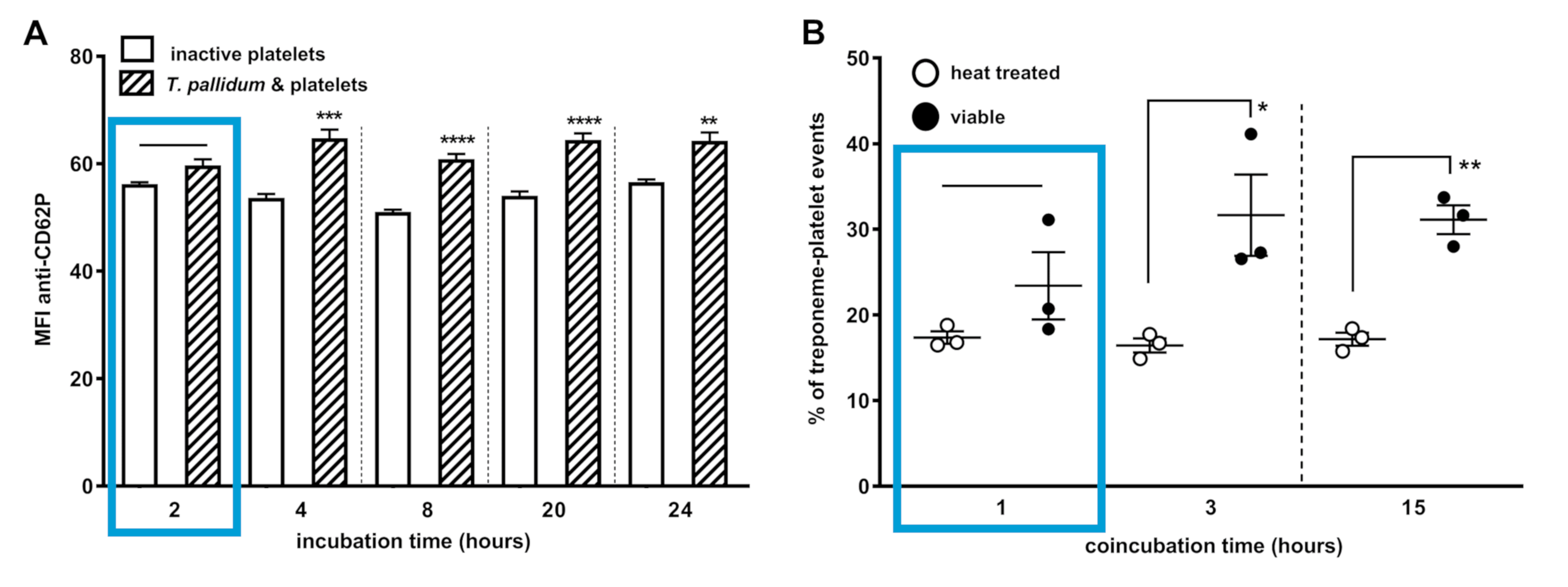

Supplement: S7 Fig — Two time course experiments demonstrate a lag period (blue boxes) for platelet activation (A) and maximal binding (B). (A) The MFI associated with the expression level of platelet activation marker CD62P was compared between initially inactive platelets (white bars) and those co-incubated with viable T. pallidum (hatched bars) after 2, 4, 8, 20 and 24 hours at 37°C. After 2 hours (blue box) there was no significant increase in CD62P up-regulation between inactive platelets (mean = 56.18 ± 0.37 [SEM], n = 4) and treponeme co-incubated platelets (mean = 59.69 ± 1.11 [SEM], n = 14). Significant CD62P expression was seen in platelets co-incubated with treponemes after 4 hours (mean = 64.77 ± 1.57 [SEM], ***P = 0.0002, n = 7), 8 hours (mean = 60.84 ± 0.95 [SEM], ****P < 0.0001, n = 5), 20 hours (mean = 64.45 ± 1.20 [SEM], ****P < 0.0001, n = 5) and 24 hours (mean = 64.23 ± 1.56 [SEM], **P = 0.0013, n = 4) compared to inactive platelets at 4 hours (mean = 53.62 ± 0.72 [SEM], n = 5), 8 hours (mean = 51.0 ± 0.42 [SEM], n = 5), 20 hours (mean = 53.98 ± 0.83 [SEM], n = 5) and 24 hours (mean = 56.56 ± 0.48 [SEM], n = 5). (B) Platelet binding was compared by flow cytometry for CFSE-labeled viable (black circles) or CSFE-labeled heat-treated treponemes (open circles) to platelets stained with PE-labeled antiCD41a after 1, 3, or 15 hours co-incubation at 37°C. After 1 hour co-incubation (blue box) there was no significant difference in platelet binding between viable (mean = 23.39 ± 3.92 [SEM], n = 3) and heat-treated (mean = 17.36 ± 0.73 [SEM], n = 3) treponemes. Viable treponemes bound significantly more platelets after 3 hours (mean = 31.65 ± 4.75 [SEM], *P = 0.0342, n = 3) and 15 hours (mean = 31.12 ± 1.67 [SEM], **P = 0.0016, n = 3) compared to heat-treated treponemes after 3 hours (mean = 16.44 ± 0.83 [SEM], n = 3) or 15 hours (mean = 17.17 ± 0.76 [SEM], n = 3). (TIF) [file pone.0210902.s007.tif]
