# Supplementary material for: Interaction of Treponema pallidum, the syphilis spirochete, with human platelets
Source: PLoS One. 2019 Jan 18;14(1):e0210902. doi: 10.1371/journal.pone.0210902 (PMC6338379; doi:10.1371/journal.pone.0210902)
Supplement: S1 Table — (DOCX) [file pone.0210902.s008.docx]

| **platelet activation state** | **inactivated** | **early activation** | **activated** | **fully activated (spread)** | **overall total** | **avg/FOV** |
| --- | --- | --- | --- | --- | --- | --- |
| **# platelets observed** | 26 | 90 | 135 | 171 | 422 | 5.6 |
| **% of total** | 6.2 | 21.3 | 32.0 | 40.5 |  |  |
| **# interactions with treponemes** | 0 | 6 | 47 | 72 | 125 | 1.7 |
| **% of total** | 0 | 4.8 | 37.6 | 57.8 |  |  |

**S1 Table**
