# Supplementary material for: Interaction of Treponema pallidum, the syphilis spirochete, with human platelets
Source: PLoS One. 2019 Jan 18;14(1):e0210902. doi: 10.1371/journal.pone.0210902 (PMC6338379; doi:10.1371/journal.pone.0210902)
Supplement: S2 Table — (DOCX) [file pone.0210902.s009.docx]

**S2 Table**

|  | **mean +/- SEM** | **mean +/- SEM** |
| --- | --- | --- |
|  | **platelet-interacting** | **non-interacting** |
| **mean ʎ wavelength (µm)** | 0.79 ± 0.02 | 0.86 ± 0.02 |
| **mean A amplitude (µm)** | 0.30 ± 0.02 | 0.26 ± 0.01 |
| **mean ʎ/A** | 2.86 ± 0.18 | 3.24 ± 0.09 |
| **mean length (µm)** | 9.56 ± 0.65 | 10.46 ± 0.66 |
| **mean axial rotation (Hz)** | 2.47 ± 0.86 | 1.58 ± 0.77 |
| **mean velocity**  **(µm/sec)**  **(µm/min)**    **N** | 1.68 ± 0.10  100.52 ± 6.18  24 | 0.65 ± 0.01  39.00 ± 5.81  15 |
| **max velocity**  **(µm/sec)**    **(µm/min)** | 2.58  154.67 | 1.7  102.08 |
| **mean acceleration**  **(µm/s^2^)** | 27.4 | 19.01 |
| **phenotype** | compact helix  rapid axial rotation  higher velocity | elongated helix  slower axial rotation  slower velocity |
