# Supplementary material for: Interaction of Treponema pallidum, the syphilis spirochete, with human platelets
Source: PLoS One. 2019 Jan 18;14(1):e0210902. doi: 10.1371/journal.pone.0210902 (PMC6338379; doi:10.1371/journal.pone.0210902)
Supplement: S3 Table — (DOCX) [file pone.0210902.s010.docx]

**S3 Table**

| **Video** |  | **Microns moved across FOV** | **sec** | **microns/sec** | **ratio**  **speed** | **ratio displacement** |
| --- | --- | --- | --- | --- | --- | --- |
| **1** | **Treponeme 1**  **Treponeme 2** | 48.64  82.98 | 13.35  9.51 | 3.64  8.73 | 2.39 | 1.71 |
| **2** | **Treponeme 1**  **Treponeme 2** | 41.92  79.80 | 28.74  25.71 | 1.46  3.10 | 2.13 | 1.90 |
| **3** | **Treponeme 1**  **Treponeme 2** | 18.19  48.70 | 31.42  31.42 | 0.58  1.55 | 2.67 | 2.68 |

**Treponeme 1 = platelet-interacting treponeme**

**Treponeme 2 = non-interacting treponeme**
