# Supplementary material for: Interaction of Treponema pallidum, the syphilis spirochete, with human platelets
Source: PLoS One. 2019 Jan 18;14(1):e0210902. doi: 10.1371/journal.pone.0210902 (PMC6338379; doi:10.1371/journal.pone.0210902)
Supplement: S4 Table — (DOCX) [file pone.0210902.s011.docx]

**S4 Table**

|  | **ratio of velocity** | **ratio of displacement** |
| --- | --- | --- |
| **Video 1** | 2.39 | 1.71 |
| **Video 2** | 2.13 | 1.90 |
| **Video 3** | 2.68 | 2.68 |
| **mean** | 2.40 | 2.10 |
| **SEM** | 0.16 | 0.30 |
| **N** | 3 | 3 |
